# Further Account of the Case of Ann Moore, in a Letter Addressed to Dr. Denman

**Published:** 1809-01

**Authors:** J. Allen

**Affiliations:** Tutbury


### *Further Account of the Case of* ANN MOORE, *in a Letter addressed to Dr.* DENMAN.

SIR,

HAD Mr. Taylor told me of his intention to send an account of Ann Moore to you for immediate publication, I might have supplied him with some further information respecting her case. But as he did not, and as I saw her daily during the time of his investigation, and made my own observations, I take the liberty of sending you the following statement, requesting you to publish it, if you think it deserving of a place, in the Medical Journal.

When I was first informed of the situation of Ann Moore, I must confess my suspicion that the report arose from some fraudulent motive; one of those wonderful histories sometimes obtruded upon the public, which on examination are found to be void of truth. But I am now convinced, that the account of her extraordinary abstinence is perfectly true; because, she could not have carried on the deception without the assistance of many other people, and because many other persons, of whose understanding and veracity there is no reason to doubt, must have been the first dupes of any imposition. But the suspicion of any intention to deceive was out of the question, by her being put under the strict watch, for sixteen days, of several persons, two at least of whom were always present with her.

Ann Moore is about 58 years of age, of a slender make and fair complexion. She has been married many years and has had several children, the youngest of which is 12 years old. I have frequently attended her, but never observed any thing peculiar in her ailments. She was subject to pulmonic complaints, but the last time I attended her, before her present illness, was in February 1805; the medicines she took were merely intended to relieve constipation of the bowels. I saw nothing of her for two years previous to this illness; nor did I ever hear a word of the story of her washing foul linen, or of her swallowing scalding hot gruel, till I read of them in Mr. Taylor's paper. These we may allow to be somewhat exaggerated by a person who does not wish to deduct from the very extraordinary circumstances of her case, but with men of sense and judgment, they will not lessen the credibility of the most important parts of the account.

When the guard was set over Ann Moore for sixteen days, for the express purpose of detecting any fallacy, if any any were practised, in the first four days she certainly took no one thing, except about two ounces of water, and in the remaining twelve days she took not a particle of any thing, either fluid or solid; nor from that time to the present fifteenth day of November, a period of eight weeks. She constantly begs not to be urged to take any thing, as the attempt to swallow gives her grievous pain, from (as she supposes) the wind in her stomach resisting its passage. Happily the poor creature has not the least inclination or appetite to eat, nor any thirst.

She has passed no fæces since the month of August, 1807, though a little wind now and then escapes. She has during that time voided her urine once in three or four days, and it has always a strong scent, and is of a high colour. Its quantity may generally be estimated at half a pint in twenty-four hours.

Ann Moore asserts, that for more than a year and a half, she has not taken any other nourishment than a little common tea without either milk or sugar; and this assertion is confirmed by the testimony of two yonng women who have lived with her.

Her countenance is fresh and animated, and her voice strong; and in the course of the sixteen days, when she was watched, I never observed her pulse to vary more than three or four strokes from 80 in a minute.

The changes produced in the external appearance of Ann Moore, seem to be just such as might be expected from mere wasting of the whole frame, particularly of the contents and integuments of the abdomen. The viscera and intestines are so shrunk as to occupy a very little space, some of them being scarcely perceptible. So are the abdominal muscles also, yet there is no reason to doubt but that they are all in their natural situation; and the ease with which the descending aorta can be felt, and sometimes seen to pulsate, is, I apprehend, to be attributed to the emaciation of those parts by which, in a state of health, it is concealed.

Early in November she had a bad cold, with violent fits of coughing, languor, a sense of weariness, and increased pain in her head, to some degree of which she is liable. Her pulse was then 116 in a minute, yet her tongue and fauces were clean and moist, and her skin soft and perspirable. She had not then voided any urine for a fortnight; and on applying my hand to the abdomen, perceiving the bladder distended, I proposed introducing the catheter; but she had no pain, and assured me she had often ten passed more than a week without voiding any urine, and did not fear but she should soon be relieved; and this accordingly happened soon after.

Now she is very much recovered from her cold, coughs seldom, and her pulse is tolerably strong, and not more than 80 in a minute.

Having related all the particulars of this, to me, unprecedented case, I must acknowledge my inability to explain them. It has been suggested, by an anatomist of high reputation, that the case of Ann Moore may probably be explained, by supposing there is some disease of the œsophagus or stomach, which prevents her swallowing. But, allowing this, physiology will have many difficulties to explain; particularly how, without any adequate support, the functions of life have been carried on. In Captain Bligh's narrative of his passage from Otaheite to Batavia, there are many circumstances related which have some affinity to the present case.

With your permission, I will from time to time send you an account of this poor woman's situation, or any material change of circumstances.

I am, &c.

J. ALLEN.

*Tutbury, December* 8, 1808.

